# Implementation evaluation of the COVID-19 vaccination: Speed, equity and mortality trends

**DOI:** 10.4102/phcfm.v18i1.5248

**Published:** 2026-07-23

**Authors:** Ilona Matthew, Michelle Viljoen, Jane McCartney

**Affiliations:** 1 School of Pharmacy, Faculty of Natural Sciences, University of the Western Cape, Cape Town, South Africa

**Keywords:** COVID-19 vaccination, speed, equity, mortality, South Africa, RE-AIM framework, Logic Model, ecological study

## Abstract

**Background:**

The coronavirus disease (COVID-19) vaccination campaign in South Africa constituted the most significant public health intervention in recent history, occurring against the backdrop of a prevailing quadruple burden of disease.

**Aim:**

To evaluate the implementation of the COVID-19 vaccination campaign, focusing on rollout speed and geographic equity and to examine the temporal associations between vaccination coverage and mortality trends.

**Setting:**

All South African residents aged 12 years and older who were eligible for COVID-19 vaccination during the study period.

**Methods:**

A retrospective, ecological population-based time-series analysis to assess vaccination rollout trends and geographic equity, and to examine the association between vaccination coverage and excess mortality. The Logic Model and the RE-AIM (Reach, Effectiveness, Adoption, Implementation and Maintenance) framework were applied to interpret implementation performance.

**Results:**

The vaccination campaign expanded rapidly in the early phases, but vaccination activity declined significantly over a three-year period (*p* < 0.001). By February 2024, approximately 49.7% of the eligible population had received at least one dose, below the national target of 67%. Coverage was higher in metropolitan than in district municipalities (*p* < 0.05). Statistically significant non-causal temporal associations were observed between vaccination coverage and excess mortality.

**Conclusion:**

Early vaccine coverage was achieved, but uptake was insufficient to meet national coverage targets. Geographic disparities suggest potential inequities in access, offering empirical insights to strengthen primary health care integration, equitable service delivery, and data-driven implementation strategies.

**Contribution:**

This study supports dual-framework implementation science for evaluating large-scale public health interventions to inform health system strengthening and emergency preparedness.

## Introduction

Coronavirus disease 2019 (COVID-19) was a worldwide public pandemic that emerged in 2019. Amid ongoing efforts to tackle prevailing health challenges in many countries, the pandemic brought healthcare needs and inequalities to the forefront.^1^ With the rapid sweep of the pandemic across the globe, it unfolded alongside ongoing health challenges. Within this global context, national vaccination programmes have demonstrated considerable variation in implementation and outcomes. In this regard, South Africa’s COVID-19 vaccination programme provides an important case for examining country-specific experiences to better understand factors influencing rollout effectiveness.

The impact of the pandemic overwhelmed the health system, both directly because of the deaths resulting from the outbreak and indirectly through heightened demand for care among patients with underlying comorbidities, resulting in additional deaths.^2^ It also highlighted the inequalities prevalent in communities and disadvantaged areas that experienced a *syndemic* pandemic, for example, where multiple pandemics or epidemics exist simultaneously.^1^

**Vaccination and health system goals:** Vaccination programmes are a key indicator of health system performance, reflecting the capacity to deliver preventive interventions equitably and at scale.^3^ Successful interventions are crucial to health services; it is essential to categorise the vulnerable and resilient indicators when prioritising resources.^4^ However, a country’s health system will only work well when its culture supports it and enough money is provided to ensure fairness of treatment to the entire population.

**Population immunity:** The South African COVID-19 vaccination campaign was implemented with two primary objectives: to reduce viral transmission and severe disease, and to ensure equitable access to vaccination for all, across diverse population groups. A national target was adopted to vaccinate 67% of the population by the end of 2021, allowing the country to achieve population immunity.^5^ Population or herd immunity refers to the protection of a population from a disease when a sufficient number of individuals are immune, either through vaccination or past infection.^6^ Based on mathematical modelling assumptions, a vaccination target of approximately 67% has been widely cited as an initial benchmark for achieving herd immunity. Mathematical modelling showed that by vaccinating 67% of the population, it would significantly reduce deaths and healthcare demand, even under conservative vaccine effectiveness scenarios.^7^ However, this threshold is highly context-dependent and varies according to factors such as viral transmissibility, population immunity and the emergence of new variants. As such, herd immunity thresholds should be interpreted as dynamic and indicative, rather than fixed targets. Accordingly, the use of a 67% coverage benchmark in this study is intended as a reference point for policy targets and not a definitive threshold for population-level immunity.

**Vaccination strategy:** The national vaccination strategy was implemented in phases, supported by the Electronic Vaccination Data System (EVDS), which enabled registration, scheduling and monitoring of vaccination uptake at the national level. Phase I prioritised healthcare workers through the Sisonke programme. Phase II expanded eligibility to high-risk populations, including older adults and individuals with comorbidities. Phase III extended access to the broader adult population.^5^ These phases reflected both epidemiological prioritisation and operational scaling of the programme. For South Africa, the COVID-19 vaccination campaign was launched in February 2021.

Several studies have examined aspects of the COVID-19 vaccination rollout in South Africa, such as vaccine supply, policy responses, vaccine hesitancy and modelling of vaccination strategies.^7^ Studies have examined vaccine manufacturing capacity, health system preparedness and operational challenges.^8^ Spatial analyses have explored geographic access to healthcare services and implications for equitable vaccine distribution.^9^ However, there remains little empirical research that evaluates the campaign across the country using real-world data and established implementation science frameworks that have simultaneously examined vaccination rollout speed, geographic equity in coverage and population-level mortality trends within a structured implementation evaluation framework.

This study addresses this gap by applying a dual-framework implementation evaluation, integrating the Logic Model and the RE-AIM framework (Reach, Effectiveness, Adoption, Implementation and Maintenance) to assess the performance of the South African COVID-19 vaccination campaign.

**Problem statement:** Despite the scale and urgency of South Africa’s COVID-19 vaccination campaign, there remains limited evidence on how effectively the intervention was implemented, particularly regarding rollout speed and geographic equity. National reporting primarily documented aggregate vaccination numbers, with little analytical insights into how the vaccination campaign performed across different regions and health system contexts, particularly among populations living outside metropolitan areas, where access to primary health care is often constrained.

In a country characterised by a persistent quadruple burden of disease and well documented inequalities, understanding implementation dynamics is critical. In addition, publicly available vaccination data seldom explore population-level trends. While national dashboards have provided routine reporting of vaccination coverage, the data have rarely been examined within a structured analytical framework linking programme implementation to broader health system outcomes. Consequently, important dimensions of programme performance remain insufficiently explored, including how the vaccination campaign unfolded over time, how equitable vaccination services were distributed across geographic municipalities and how vaccination coverage trends corresponded with national mortality patterns during the pandemic. Addressing the gap requires systematic evaluation of the vaccination campaign using implementation science approaches to contextualise programme performance within the broader health system.

### Aim

The study aimed to evaluate the South African COVID-19 vaccination campaign using a quantitative ecological time-series approach. The analysis examined rollout speed, geographic equity in vaccination coverage and temporal associations between vaccination coverage and mortality trends using the Logic Model and the RE-AIM implementation framework to interpret implementation performance across the country.^10,11^

**Research questions:** *How rapidly and equitably was the South African COVID-19 vaccination implemented, and what temporal association between vaccination coverage and mortality trends can be observed when using the RE-AIM and Logic Model implementation frameworks?*

Secondary questions

- **Rollout speed:** How did the vaccination uptake and rollout speed change over the course of the national vaccination campaign?
- **Geographic equity:** Were there statistically significant differences in vaccination coverage between metropolitan and district municipalities?
- **Implementation performance:** How did the Logic Model components and the RE-AIM domains explain the implementation performance of the campaign in terms of Reach, Adoption, Effectiveness, Implementation and Maintenance?

By integrating national vaccination, geographic coverage and mortality surveillance with the Logic Model and the RE-AIM implementation framework, this study provides a framework-based evaluation of the South African COVID-19 vaccination campaign and contributes implementation evidence on how large-scale public interventions can be assessed in terms of speed, equity and health system performance in resource-constrained settings.

## Research methods and design

### Study design

The study employed a retrospective ecological time-series design using aggregated national- and municipal-level data to evaluate the implementation of the COVID-19 vaccination campaign in South Africa. The analysis covered the period from February 2021 to February 2024 and examined the speed of the vaccination rollout, geographic equity in coverage and mortality trends. As an ecological study, all analyses were conducted at population level, without individual linkage, and findings are therefore interpreted as non-causal associations.

The study examined vaccination uptake over time, geographic distribution of vaccination coverage across the country and the mortality patterns that were observed during the pandemic period. The primary outcomes included: (1) vaccination uptake rates of the population over time, (2) equity of access as shown by the geographic coverage, (3) excess and all-cause mortality trends and (4) temporal associations between vaccination coverage and mortality.

### Conceptual frameworks

**FIGURE 1:**
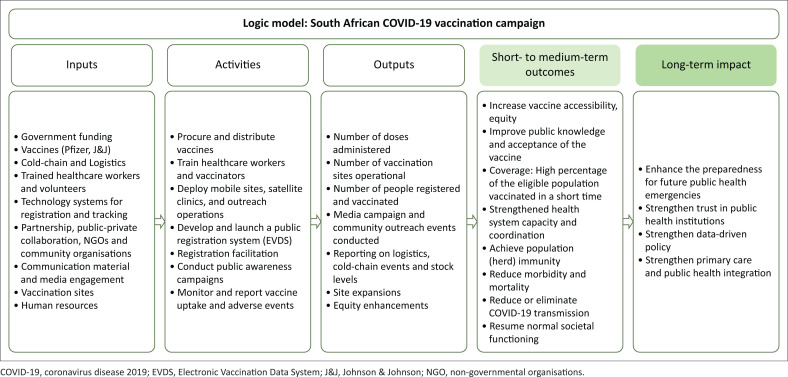
Logic Model of the coronavirus disease 2019 vaccination campaign in South Africa. The figure illustrates the sequential relationship between programme inputs, implementation activities and outputs, and their linkage to short- to medium-term outcomes and long-term public health impacts.

Two complementary frameworks guided the implementation evaluation. The Logic Model was used to map the vaccination programme across inputs, activities, outputs, outcomes and impact, providing a structured representation of implementation processes and intended effects.^10^ The model has two sides: process and outcome. The process component described the resources and infrastructure that supported the vaccination campaign, the programme activities such as the vaccine delivery and the outputs, including vaccination coverage. The outcome component examined short and medium-term effects and shows the results the programme aims for, which can occur within a short period or over a longer time. Figure 1 illustrates the Logic Model’s relationship of activities throughout the vaccination campaign.

**FIGURE 2:**
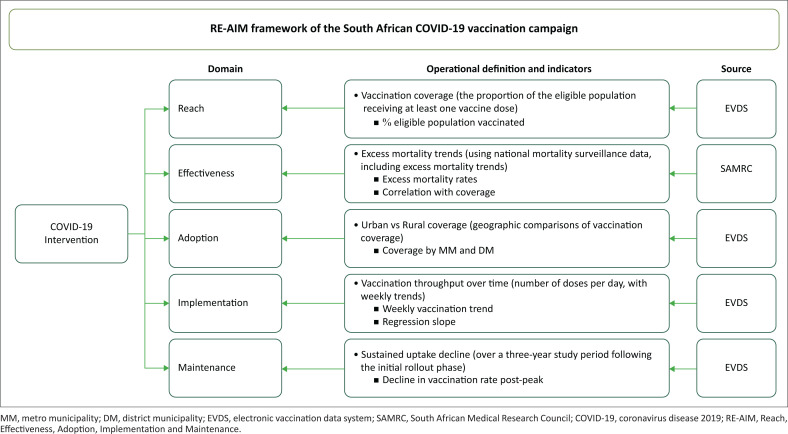
Operationalisation of the Reach, Effectiveness, Adoption, Implementation and Maintenance framework in the South African coronavirus disease 2019 vaccination campaign.^11^ Each domain is linked to specific operational definitions and measurable indicators, including vaccination coverage, excess mortality trends, geographic disparities (urban vs. rural), vaccination throughput over time and sustained uptake patterns.

The RE-AIM framework was then applied to evaluate the evidence-based intervention.^11^ To operationalise the RE-AIM framework in this study, measurable indicators were defined for each domain using the available national datasets, as summarised in Figure 2. *Reach* was assessed using national vaccination coverage. *Adoption* was evaluated through geographic comparisons of vaccination coverage. *Implementation* was assessed through vaccination throughput over time. *Effectiveness* was examined using national mortality surveillance data during the pandemic period. *Maintenance* was evaluated through sustained vaccination uptake over the 3-year study period. These indicators enabled the application of the RE-AIM framework to population-level outcomes using aggregated national data. For this research, the framework enabled a structured evaluation of how widely the COVID-19 vaccination campaign has reached the population, how effectively it was implemented and whether it was sustained over time.

### Study setting and population

The study population comprised all South African residents aged 12 years and older who were eligible for COVID-19 vaccination during the study period. Eligibility expanded during Phase II of the rollout (September 2021) to include individuals aged 12–17 years, resulting in a final eligible population of approximately 46 million.

For the purposes of this study, key terms were defined. Vaccination coverage refers to the proportion of the eligible population that received at least one dose of the COVID-19 vaccine. Vaccination uptake refers to the number of vaccines administered over time. Reach, as defined within the RE-AIM framework, refers to the extent to which the target population was exposed to or received the intervention, operationalised in this study using vaccination coverage across geographic areas. These terms are consistently used throughout the manuscript to distinguish between vaccination volume, population coverage and implementation reach.

### Inclusion criteria

The campaign data included vaccinations for the South African population, 12 years and over. Only vaccination data that were captured on the live EVDS were included. Vaccination data covered the period from 17 February 2021 to 29 February 2024, while mortality data covered the period from 2015 to 2023, allowing for the construction of the pre-pandemic baseline.

### Exclusion criteria

Data points not captured in the EVDS were excluded from the analysis, and analyses were restricted to recorded and validated entries in the national datasets. The dataset excludes unreported deaths. The study excludes individuals less than 12 years of age.

### Data sources and collection

Secondary data were obtained from publicly available sources. Population estimates (2015–2023) were sourced from the World Bank Group and cross-verified by Worldometer.^12,13^ Vaccination data were obtained from the National Department of Health (NDoH) of South Africa, covering the period from 17 February 2021 to 29 February 2024.^14^ Vaccination data were collated over 3 years, from the start of the vaccination campaign period, defined as 17 February 2021, to the end of February 2024, which corresponds to the national vaccine rollout following programme initiation. Mortality data were obtained from the South African Medical Research Council (SAMRC), including observed and expected deaths, from 2015 to 2023.^15^ Confirmed COVID-19 mortality data were obtained from Our World in Data to support contextual interpretation of mortality trends during the pandemic.^16^ The pre-vaccination baseline period was defined as 01 March 2020 – 16 February 2021, prior to the introduction of the COVID-19 vaccine in South Africa.

**Analytical dataset:** The dataset comprised weekly vaccination counts, municipal vaccination coverage and national mortality data spanning February 2021 – February 2024. Mortality baseline estimates were derived from weekly mortality surveillance data for the period 2015–2019.

**Data preparation:** Datasets were reviewed for completeness, consistency and alignment across the time periods. Vaccination and mortality data were harmonised using epidemiological weeks to ensure temporal comparability. All variables were standardised to ensure consistency in units of measurement and population denominators across the datasets. Vaccination data were aggregated by epidemiological week and converted to cumulative vaccination coverage, allowing linkage of vaccination coverage and mortality trends over time. Mortality datasets were organised into weekly observed deaths, and expected deaths were calculated from the 2015 to 2019 baseline period.

**Excess mortality was calculated as:** Excess deaths = observed deaths – expected deaths.

These calculations enabled the identification of deviations from expected mortality during the pandemic period. No imputation of missing data was performed. Data points were assessed and retained only if consistent with overall reporting trends, as the datasets were derived from national surveillance systems. Data processing steps were conducted systematically to enable reproducibility of the analysis using publicly available datasets.

#### Operationalisation of geographic equity

Geographic equity was operationalised as differences in vaccination coverage between metropolitan municipalities (urban) and district municipalities (predominantly rural or mixed), based on the NDoH classifications. This approach reflects spatial access to vaccination services and serves as a proxy for urban-rural differences. The analysis does not include socioeconomic stratification or direct measures of access.

#### Statistical analysis

All analyses were conducted using Microsoft Excel, which supports regression and correlation analyses suitable for exploratory evaluation of ecological time series. Vaccination and mortality datasets were cleaned and harmonised. Descriptive analyses were conducted to summarise vaccination uptake, coverage trend and mortality patterns over time. Inferential statistics were performed and included:

- Linear regression to assess the trends in weekly vaccination uptake over time (rollout speed).
- Segmented (interrupted time-series) analysis to identify changes in vaccination trends across the implementation phases.
- Independent-sample *t*-tests to compare differences in vaccination coverage between metropolitan and district municipalities (geographic equity).
- Pearson correlation analyses to assess associations between vaccination coverage and excess mortality.
- Lagged regression analyses (3- and 6-week lags) to examine delayed temporal associations between vaccination coverage and mortality.^17,18^

Statistical significance was assessed at a two-sided alpha level of 0.05, and confidence interval (CI) ranges were reported for key estimates. The analyses assumed approximate linearity and normality of residuals. No adjustments were made for time-varying confounders, including variant dynamics, prior infection and non-pharmaceutical interventions because of the limitations in the aggregated dataset. Accordingly, the results represent unadjusted population-level associations and should be interpreted as exploratory rather than causal.

#### Analytical approach

The analysis followed a structured sequence: (1) data preparation and harmonisation, (2) descriptive assessment of vaccination rollout, (3) inferential analysis of rollout speed, geographic equity and mortality associations, and (4) interpretation using the Logic Model and the RE-AIM implementation frameworks to assess programme performance in terms of speed, equity and effectiveness.

Rollout speed was assessed through regression-based time-series analysis of weekly vaccination counts. Geographic equity was evaluated using group comparisons of coverage across municipal classifications. Mortality trends were analysed using excess mortality estimates and correlated with vaccination coverage, including lagged analysis to account for delayed population-level effects, following vaccination. All findings were interpreted within the constraints of an ecological study design and do not imply causality.

### Ethical considerations

This article followed all ethical standards for research without direct contact with human or animal subjects.

## Results

This section presents the study’s findings, providing insight into South Africa’s COVID-19 vaccination campaign. The results are presented to evaluate changes in vaccination over time (speed), geographic differences in coverage (equity) and temporal associations between vaccination coverage and mortality, using both descriptive and inferential analyses, in alignment with the study objectives.

### Population characteristics

**FIGURE 3:**
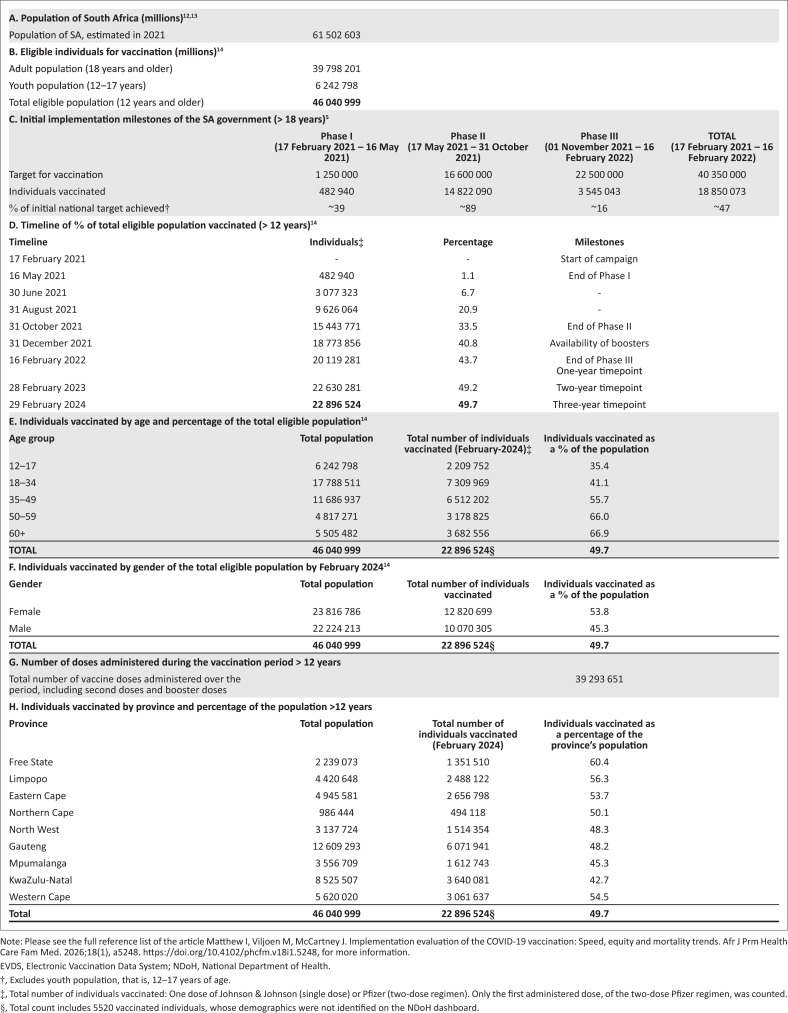
Overview of the South African coronavirus disease 2019 vaccination campaign data.

The population estimates indicated that South Africa had approximately 61.5 million individuals in 2021, of whom 46 040 999 million were eligible for the COVID-19 vaccination once the 12–17 years age group was included in the campaign. These figures formed the denominator for evaluating the vaccination coverage (Figure 3).

### Vaccination rollout and programme reach (Logic Model: *Outputs* and RE-AIM: *Reach*)

The national vaccination campaign began on 17 February 2021, with a phased-in approach, prioritising healthcare workers and the high-risk population groups. During Phase I, approximately 39% of the targeted healthcare workforce were vaccinated within the designated time frame. During Phase II, the eligibility was expanded to older adults and essential workers, and the campaign achieved approximately 89% of its population target. Phase III further expanded eligibility to all adults. Approximately 16% of the phase target was achieved during the predetermined period (Figure 3).

By February 2022, approximately 43% of the eligible population had received at least one dose of the vaccine. By February 2024, cumulative coverage reached approximately 49.7% of the eligible population. Vaccination coverage varied across provinces, ranging from 42.7% in KwaZulu-Natal to 60.4% in the Free State. These values represent cumulative coverage at the end of the study period. Within the Logic Model framework, these outputs represent the direct implementation results of the vaccination campaign. Within the RE-AIM framework, they reflect the programme reach, defined as the proportion of the target (eligible) population receiving this intervention. Figure 3 summarises key demographic, implementation and vaccination indicators for the national COVID-19 vaccination programme. It includes population, targets across phases and temporal trends in vaccine coverage over 3 years, stratified by age, gender, province and dose.

### Vaccination throughput and Implementation dynamics (Logic Model: *Activities* and RE-AIM: *Implementation*)

An analysis of vaccination throughput shows it has declined substantially over time. During the first year of the programme, an average of 54 896 doses were administered per day (20 201 628 doses over 368 days). This decreased to 6599 doses per day in the second year and to 808 doses per day in the third year.

This decline in vaccination throughput within the Logic Model reflects changes to the programme’s activity levels, while within RE-AIM, it reflects variation in implementation fidelity and maintenance over time.

### Time-series analysis of vaccination rollout speed

**FIGURE 4:**
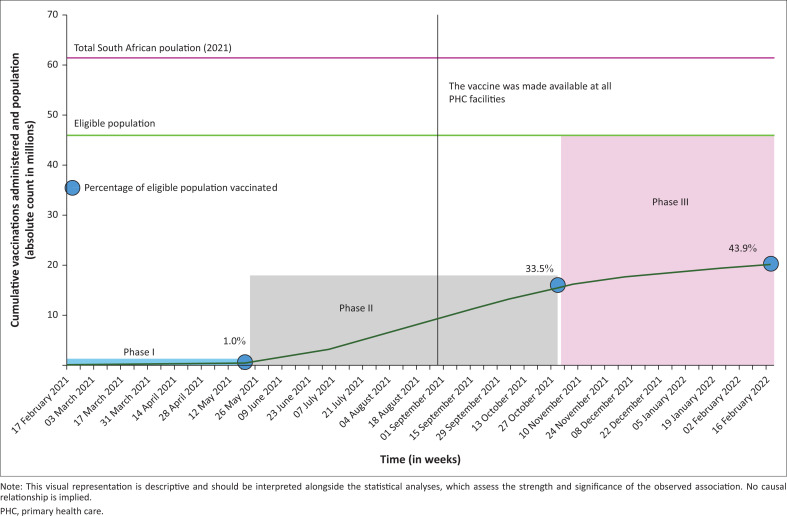
Weekly coronavirus disease 2019 vaccination uptake over time, illustrating the temporal trend in vaccination and coverage from February 2021 to February 2022, with phases and milestones.

Linear regression analysis demonstrated a statistically significant decline in weekly vaccination uptake over time. The regression model estimated: *Vaccinations_t_* = *β*_0_ + *β*_1_ *(Time)*. The intercept (*β*_0_) was 399 625.16 (*p* = 2.83 × 10^–26^). The slope coefficient for time (*β*_1_) was –3137.32 (95% CI: –3839 to –2507, *p* < 0.001), indicating a reduction of approximately 3137 vaccinations per epidemiological week following the initial rollout phase. As illustrated in Figure 4, weekly vaccination counts decreased following the initial peak of the rollout. This graphical trend is descriptive and reflects the temporal pattern of uptake. Inferential conclusions regarding the rate of decline are based on the regression analysis rather than the visual representation alone.

### Geographic distribution and equity in vaccination coverage (Logic Model: *Short-term outcomes* and RE-AIM: *Adoption*)

Equity is a multidimensional construct encompassing social, economic and structural determinants that were not fully captured in the analysis due to data constraints. Equity was operationalised as geographical equity in vaccination coverage, assessed by municipal classification as a proxy for urban-rural differences. This approach reflects spatial access to vaccination services but does not capture socioeconomic status or individual-level determinants.

To evaluate geographic equity, vaccination coverage was compared between metropolitan municipalities (MM), which represented mostly urban settings, and district municipalities (DM), which represented mostly rural or mixed rural settings. Vaccination coverage varied across provinces: 42.7% (in KwaZulu-Natal) to 60.4% (in the Free State) of the eligible population. Provinces such as the Free State (60.4%), Limpopo (56.3%) and the Western Cape (54.5%) achieved higher coverage levels than other regions (Figure 3).

Using the national population as the denominator, vaccination coverage was significantly higher in metropolitan municipalities than in district municipalities across all years, with a combined urban mean of 0.0091 (95% CI: 0.0032 to 0.0150) compared to a rural mean of 0.0021 (95% CI: 0.0016 to 0.0026), with *p* < 0.05. Similar patterns were observed when using provincial population denominators.

However, when coverage was calculated using district-level population denominators, no statistically significant differences were observed between metropolitan and district municipalities in any year or in the combined analysis (*p* > 0.05) (Online Appendix 1).

Within the RE-AIM framework, these results represent differences in programme adoption across geographic settings. Within the Logic Model, they represent the disparities in programme outcomes related to service accessibility.

### Mortality trends (Logic Model: *Intermediate outcomes* and Re-AIM: *Effectiveness*)

The SAMRC maintained ongoing surveillance of national mortality by providing weekly reports on excess natural deaths during the COVID-19 pandemic.^15^ Historical mortality data between 2015 and 2019 were approximately 528 548 deaths per year, equivalent to approximately 10 164 deaths per week under non-pandemic conditions. During the pandemic period, mortality levels deviated from this baseline. The largest increase in deaths occurred during 2021, with 705 354 deaths recorded. Table 1 presents national population estimates alongside all-cause mortality, predicted deaths, confirmed COVID-19 deaths and calculated excess mortality for the period 2015–2023.

**TABLE 1:** Annual population estimates and mortality trends in South Africa (2015 to 2023), emphasising mortality data in 2021.

Year	Population^13^	Mortality^15^ (all-cause deaths)	Predicted deaths^15^	Confirmed COVID-19 deaths^16^	Excess deaths (minus COVID-19 deaths)^15^
2019	59 587 828	528 548†	-	-	-
2020	60 562 321	588 952	532 428	28 033	28 491
2021‡	61 502 553	705 354	513 670	63 028	128 656
2022	62 378 377	555 024	509 059	11 507	34 458
2023	63 212 339	544 359	510 302	27	34 030

Note: Please see the full reference list of the article Matthew I, Viljoen M, McCartney J. Implementation evaluation of the COVID-19 vaccination: Speed, equity and mortality trends. Afr J Prm Health Care Fam Med. 2026;18(1), a5248. https://doi.org/10.4102/phcfm.v18i1.5248, for more information.

COVID-19, coronavirus disease 2019.

† , The average of annual mortality data from 2015 to 2019;

‡ , Emphasising the mortality data for 2021 as significant.

**FIGURE 5:**
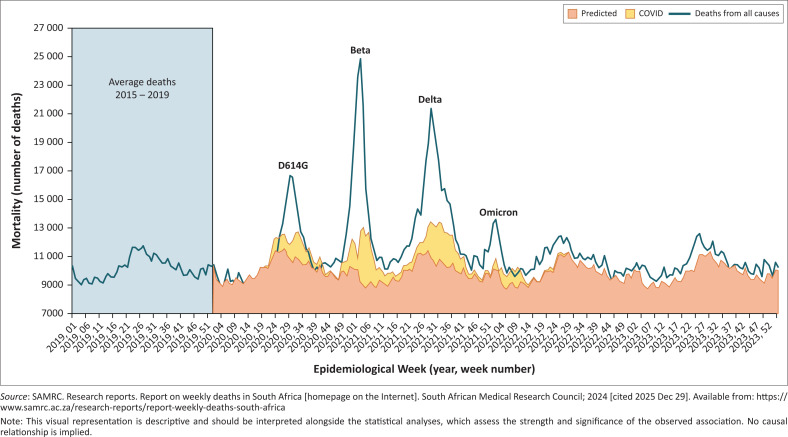
Temporal trends in all-cause mortality, compared to predicted deaths and reported coronavirus disease 2019 deaths and the different variant waves, across the study period.^15,16^

The data illustrate that weekly excess mortality peaked in mid-2021, while vaccination coverage remained below 25% of the eligible adult population. Following the expansion of the vaccination coverage during late 2021 and early 2022, data show that excess mortality began to decline, and mortality levels moved closer to expected baseline values (Table 1, Figure 5). Within the RE-AIM framework, these mortality trends represent programme effectiveness at the population level.

### Association between vaccination coverage and mortality

Pearson correlation analysis was conducted to evaluate the association between vaccination coverage and excess mortality. The contemporaneous analysis demonstrated a moderately strong positive correlation between vaccination coverage and excess mortality, with *r* = 0.6819 (95% CI: 0.5884 to 0.7574) and *p* < 0.05. Figure 5 illustrates the co-occurring trends in variant waves and mortality over time. While the graphical representation suggests an inverse temporal pattern, it is descriptive in nature and should be interpreted alongside statistical analyses.

A lagged regression analysis examines whether changes in vaccination coverage are associated with subsequent changes in mortality. To account for potential delayed effects of vaccination and to further examine these temporal relationships, linear regression models were estimated using vaccination coverage variables. Lagged analysis showed a decreasing correlation strength with increasing lag duration. Table 2 presents Pearson correlation coefficients assessing the temporal associations between vaccination coverage and excess mortality under different lag conditions.^17,18^

**TABLE 2:** Lagged correlation analysis between vaccination coverage and excess mortality.

Lag window	Correlation (*r*)	Interpretation
No lag (as is)	0.6819	There is a strong contemporaneous association between vaccination coverage and excess mortality trends.
3-week lag (severe disease reduction in hospital and ICU	0.5532	There is a moderate association when coverage is shifted by 3 weeks relative to the mortality data.
6-week lag (population level impact)	0.3851	There is a weaker association when vaccination coverage is shifted by 6 weeks.

ICU, intensive care unit.

## Discussion

The findings of this study evaluate the implementation performance of the COVID-19 vaccination campaign, interpreting them in relation to existing evidence and using them to inform context-specific recommendations. The evaluation integrates empirical findings with implementation science frameworks to interpret rollout dynamics, geographic equity and population-level mortality trends.

### Synthesis of key findings

The vaccination campaign achieved rapid initial expansion but was not sustained over time. A statistically significant decline in vaccination activity was observed. Linear regression analysis showed that weekly vaccinations decreased by approximately 3173 doses per epidemiological week. This pattern indicates that the campaign’s momentum declined after the early rollout phase and suggests that even though implementation capacity enabled rapid deployment, sustaining the vaccination demand and programme intensity proved challenging.

By February 2024, the cumulative national vaccination coverage reached 49.7% of the eligible population. When interpreted relative to the national implementation targets and phased rollout milestones, including the benchmark of approximately 67% population coverage, the initial observed vaccination throughput during the first year (43.7%) indicates rapid early deployment of the vaccination programme. However, the pace was insufficient, as the subsequent declines in daily vaccination rates in the second (49.2%) and third (49.7%) years did not meet the sustained coverage targets within the projected timeframe. This demonstrates a partial reach of the intervention.

The observed inter-provincial variation in vaccination coverage, ranging from 42.7% to 60.4%, indicates heterogeneity in implementation rollout performance across regions, although the magnitude of variation was moderate. These findings suggest that early rollout success did not translate into sustained population-level coverage.

Geographic equity analysis showed higher vaccination coverage in metropolitan municipalities than in district municipalities when assessed using national and provincial denominators (*p* < 0.05). However, differences were not statistically significant when district-level population denominators were applied, and absolute differences were modest. This indicates that, while spatial disparities were present, their magnitude was limited, highlighting the importance of interpreting both statistical and practical significance in equity analyses. The use of municipal classification as a proxy for access further indicates that these findings reflect geographic rather than socioeconomic equity. The findings suggest that health system infrastructure, service accessibility and factors such as vaccine hesitancy or demand-related barriers may have influenced the distribution and uptake of vaccination services.

Mortality trends showed that excess mortality peaked during the early epidemic waves of 2021, when vaccination coverage remained quite low and declined following the expansion of vaccination coverage. However, these trends represent population-level temporal patterns and cannot be attributed solely to vaccination. Multiple concurrent factors likely influenced mortality dynamics, including epidemic waves, variant evolution, prior infection and hybrid immunity, improvements in clinical management, and public health non-pharmaceutical interventions such as lockdowns and mask mandates. These factors likely interacted dynamically during the pandemic and may have influenced mortality patterns independently or synergistically.

Statistical analysis identified moderate temporal associations between vaccination coverage and excess mortality trends. Lagged correlation analyses provided additional insight into the temporal relationship between vaccination coverage and excess mortality. Pearson correlation analysis showed a contemporaneous correlation coefficient of *r* = 0.6819, with decreasing correlation strength observed in lagged analyses. The strongest correlation was observed when vaccination coverage and excess mortality were analysed contemporaneously, while the strength of the association declined when vaccination coverage was shifted forward by 3 weeks and 6 weeks. The reported CIs provide an estimate of the precision of the observed associations and should be considered when interpreting the strength and reliability of the findings.

These findings indicate that the relationship between vaccination coverage and mortality varied over time and was not temporally synchronised in a simple linear manner. Given the ecological design and a lack of adjustment for time-varying confounders, this regression model quantifies statistical associations with the observed time-series data and should be interpreted as non-causal. In addition, these observed findings are based on aggregated national population-level data without individual-level linkage between vaccination coverage, vaccination status and mortality outcomes, limiting causal interpretation. The findings should be interpreted as population-level temporal associations rather than evidence of causal effects.

Collectively, the results demonstrate that the vaccination campaign expanded rapidly but did not sustain sufficient uptake to achieve national coverage targets. At the same time, geographic disparities in coverage and the observed temporal association with mortality trends provide insight into how implementation performance varied and evolved across the health system.

### Interpretation using the Logic Model and aligned outcomes

For an intervention to be effective, planning and execution are vital. The Logic Model provided a structured lens to interpret the implementation pathway and assessed the outcomes and impact of the campaign, as it focused on equity, access, health system coordination and improving the public knowledge and their acceptance (Figure 1).

At the level of inputs and activities, South Africa mobilised substantial infrastructure, including public–private partnerships, mass vaccination sites and the EVDS.^19^ Operational strategies such as phased prioritisation, mobile vaccination units, outreach programmes and extended service hours facilitated early access and rapid scale-up.^20,21^ These measures reflect the resources and operational strategies deployed to support vaccine delivery. However, implementation challenges, including coordination constraints, workforce pressures and limited rural integration, likely influenced the programme performance.

The outputs of the intervention were reflected in vaccination coverage and programme reach. Although the campaign achieved early expansion and vaccinated more than 20 million individuals within the first year, overall national coverage plateaued below 50%. The plateau observed in Phase III suggests that the operational capacity alone was insufficient to sustain demand once the high-risk populations had been vaccinated.

At the level of short- and medium-term outcomes, improvements in geographic access were observed through the integration with primary health care services and outreach programmes. However, spatial variation in access persisted.^9^ Changes in mortality patterns coincided temporally with increased vaccination coverage, but the ecological design of the study limits the ability to attribute these changes directly to vaccination.

### Interpretation using the RE-AIM framework

The RE-AIM framework was operationalised using measurable indicators aligned with each domain, as summarised in Figure 2. Metrics were used to evaluate the vaccination campaign across reach, effectiveness, adoption and implementation, and it enabled a multidimensional evaluation of the implementation performance (Figure 2):

- **Reach:** The programme achieved coverage of approximately 49.7% of the eligible population, indicating substantial but incomplete population reach. Variation across provinces reflects uneven distribution of programme coverage.
- **Effectiveness:** Population-level mortality declined following the expansion of the vaccination coverage, consistent with broader evidence on vaccine impact.^7^ However, these findings reflect aggregated temporal patterns and because of the ecological nature of the analysis, the study cannot isolate vaccination as the sole driver of mortality reductions.
- **Adoption:** Equity is reflected in this domain, representing the extent to which the intervention reached different geographic populations. Vaccination services were widely implemented across healthcare settings, including primary health care facilities, hospitals, pharmacies and outreach platforms. The integration of COVID-19 vaccination services into routine primary health care services represented an important operational adaptation, designed to increase access.^22^ This decentralisation facilitated point-of-care delivery of vaccines during routine service encounters. Geographic variation in coverage suggests differential adoption across regions, influenced by access and system-level factors.
- **Implementation:** The phased rollout strategy enabled rapid early deployment. However, the decline in vaccination throughput indicates challenges in maintaining programme intensity, potentially reflecting reduced perceived risk, demand saturation among high-risk groups and behavioural factors such as vaccine hesitancy.
- **Maintenance:** Sustained uptake declined over time, despite continued availability of vaccination and booster programmes. This suggests that long-term maintenance of vaccination programmes requires ongoing public engagement, risk communication and integration into routine healthcare delivery.

### Comparison with existing literature

The observed decline in vaccination uptake over time is consistent with international evidence demonstrating reduced demand for COVID-19 vaccination following the initial rollout phases.^23,24^ This pattern reflects the saturation of high-risk populations and the increasing reliance on voluntary uptake among lower-risk groups.

Geographic disparities in vaccination coverage have also been widely reported across countries. The observed urban-rural disparities are consistent with the literature from both high-income and middle-income settings, which demonstrate that urban populations typically achieve higher vaccination coverage than rural populations, reflecting differences in healthcare infrastructure, geographic access and service delivery.^25^ These structural determinants of healthcare access are particularly relevant in South Africa, where spatial inequities in health system resources have been well documented.^26^

The mortality trends observed in this study align with broader global evidence, demonstrating that COVID-19 vaccination programmes contributed to reductions in hospitalisation and mortality at the population level.^27^ However, several additional factors may also have influenced mortality patterns during the pandemic, including improvements in clinical management, changes in variant virulence, prior infection-induced immunity and non-pharmaceutical interventions.^28^

### Implications and recommendations

The findings of this study provide important insights for strengthening the implementation of large-scale public health interventions, particularly in resource-constrained and inequitable health system contexts. The observed trajectory of the COVID-19 vaccination campaign in South Africa highlights the distinction between rapid programme initiation and sustained implementation performance, with implications for future emergency preparedness and health system resilience.

A central implication relates to the sustainability of intervention uptake. While the vaccination campaign achieved rapid early expansion, the subsequent decline in vaccination activity highlights that implementation capacity alone is insufficient to maintain programme momentum. Sustained uptake requires continuous demand-generation strategies that address behavioural, structural and contextual determinants of health service utilisation. Targeted risk communication, community engagement and context-specific interventions to address vaccine hesitancy and perceived risk are essential to maintaining programme effectiveness beyond the initial rollout phase.

Geographic equity remains a critical consideration for public health implementation. The observed differences between metropolitan and district municipalities suggest that structural factors, such as health system infrastructure, service accessibility, and resource distribution, continue to influence the reach of national interventions. Therefore, strengthening equitable access requires targeted investment in non-metropolitan areas, including expansion of mobile outreach services, reinforcement of community-based healthcare delivery and improved integration of services at primary health care level. These strategies are particularly important in settings characterised by persistent spatial and socioeconomic inequalities.

The COVID-19 vaccine was not immediately available at primary health care facilities during the initial rollout. The integration of vaccination services into routine primary health care represents a key implementation strategy for enhancing both equity and sustainability. Embedding preventive interventions within existing care platforms, such as chronic disease management, maternal and child health services, and community-based care, can reduce access barriers, improve continuity of care and support more resilient health system responses during public health emergencies. This approach aligns with broader principles of health system strengthening, emphasising integration, accessibility and continuity of care.

From a systems perspective, the findings highlight the critical role of data-driven decision-making in public health implementation. The EVDS enabled real-time monitoring of vaccination coverage and programme performance. However, the absence of integrated linkage between vaccination data, mortality surveillance and clinical datasets limits the ability to fully evaluate intervention outcomes. Strengthening integrated health information systems to enable interoperability across data sources would support more precise, timely and context-specific policy responses.^29^ Such systems would allow for the identification of underserved populations, monitoring of geographic disparities and evaluation of intervention effectiveness in real time, thereby enhancing both responsiveness and accountability.

More broadly, the application of the Logic Model and RE-AIM frameworks demonstrates the value of structured implementation science approaches in evaluating complex public health interventions. The integration of these frameworks enables a link between programme inputs, processes and measurable outcomes, facilitating a more comprehensive understanding of implementation performance. Embedding such frameworks within routine programme evaluation can strengthen the translation of empirical findings into actionable policy and improve the design, monitoring and adaptation of future interventions.

Collectively, these findings emphasise that effective public health interventions require not only rapid deployment but also sustained engagement, equitable access, integrated service delivery and robust data systems. Strengthening these dimensions will be critical for improving the implementation of future vaccination programmes and enhancing preparedness for emerging public health threats.

### Limitations

The study has several important limitations. Firstly, the ecological study design, based on aggregated population-level data, precludes causal inference and introduces the potential for ecological fallacy. Associations observed at the population level may not reflect individual-level relationships. Secondly, vaccination coverage and mortality data were not linked at the individual level, limiting the ability to directly assess vaccine effectiveness. Thirdly, the analysis did not adjust for key time-varying confounding factors, including variant dynamics, prior infection and hybrid immunity, changes in clinical management and non-pharmaceutical interventions, all of which may have influenced mortality trends independently of vaccination coverage. Fourthly, the equity analysis was limited to geographic comparisons using municipal classifications as a proxy for urban-rural differences. It also did not include socioeconomic stratification or formal equity indices, thereby providing only a partial assessment of equity. Fifthly, the use of secondary administrative datasets may be subject to reporting delays, data completeness issues and potential inconsistencies across sources. Sixthly, behavioural factors, including vaccine hesitancy, risk perception and public perception, were not measured in this study, yet may have played a significant role in shaping vaccination uptake and observed coverage patterns. Finally, while time-series regression analyses were conducted, more advanced modelling approaches, such as fully specified interrupted time-series analyses or adjustment for autocorrelation, were not applied. The findings should therefore be interpreted as exploratory population-level associations rather than definitive estimates of effect. Despite these limitations, the study provides valuable population-level insights into vaccination rollout dynamics within a real-world public health context.

## Conclusion

The study provides a population-level implementation evaluation of the COVID-19 vaccination programme in South Africa. While the initial rollout achieved high vaccination uptake, momentum declined over time, and geographic disparities in coverage were observed. A temporal association between vaccination coverage and reduced mortality was identified, although causality cannot be inferred. These findings highlight the importance of sustained implementation strategies, equitable service delivery and data-driven monitoring to support future public health interventions.
